# Prehospital and Emergency Department Capacity for Acute Stroke Care in Minnesota

**Published:** 2008-03-15

**Authors:** Albert Wu Tsai

**Affiliations:** Minnesota Department of Health

## Abstract

**Introduction:**

Stroke is the third leading cause of death in Minnesota. One strategy to reduce the burden of stroke is to implement systems-level improvements in the prehospital and acute care settings. Two surveys conducted in 2006 obtained information about current practices and capacities of emergency medical services and emergency departments in Minnesota.

**Methods:**

In 2006, the Minnesota Department of Health and the Minnesota Stroke Partnership (the statewide stroke collaborative group) conducted two surveys. The survey for emergency medical services organizations, mailed to every licensed ambulance service in Minnesota, asked about transportation policies and training needs. The survey for hospitals, mailed to every hospital in the state, asked about capacity to treat acute stroke. Results were calculated using simple frequency analyses.

**Results:**

Of 257 surveys mailed to ambulance services, 199 (77%) were returned. Ambulance services generally considered stroke an emergency. Training on stroke was reported most effective in person annually or semiannually. Of 133 surveys mailed to hospitals, 120 (90%) were returned. Stroke capacity differed markedly between hospitals in rural areas and hospitals in the large Minneapolis–St. Paul metropolitan area. Many hospitals, particularly small hospitals, reported lacking stroke protocols. Training for stroke is needed overall but particularly in small hospitals.

**Conclusion:**

Transport and treatment of people with acute stroke in Minnesota vary by hospital size and location. Standardization of transport and protocols for acute treatment may increase efficiency and overall care for stroke patients. In addition, the need to train ambulance personnel and emergency departments about stroke remains high.

## Introduction

In the United States, an estimated 700,000 strokes occur annually (1). Although Minnesota is consistently rated one of the healthiest states in the nation (2), stroke is a major cause of death and disability for Minnesotans. In 2005, 2372 people died from stroke, making it the third leading cause of death in the state (3). In addition, approximately 11,000 hospitalizations for stroke occur annually, with estimated costs exceeding $266 million (Minnesota Department of Health, Minnesota Hospital Discharge Dataset, unpublished data, 2005).

The American Heart Association recently released recommendations for comprehensive systems of stroke care (4). These include establishing processes to provide rapid access to emergency medical services (EMS) for people with acute stroke and promoting the use of transport and treatment protocols by EMS personnel. In addition, hospital care for acute stroke should include strategies to ensure appropriate and efficient triage and treatment of stroke patients and use of clinical pathways (i.e., procedures and protocols outlined through a decision tree, usually provided on a paper document attached to a medical chart). The Minnesota Stroke Partnership (MSP) — a coalition of stroke care professionals, advocates, and others interested in stroke prevention and treatment representing various health care delivery organizations in Minnesota — considers prehospital transport and treatment and emergency care as priority systems. MSP works with the Minnesota Department of Health (MDH) to plan and implement systems-level changes. Because these systems had not been formally assessed to determine gaps or needs, MSP and MDH conducted two surveys to assess the preparedness and capacity of these systems.

## Methods

### Minnesota Emergency Medical Services Stroke Care Survey

A survey of EMS directors was developed in 2006 with input from MSP members to collect information about prehospital stroke care capacity and practices among Minnesota ambulance services. The survey also provided information to MSP about stroke training needs and effective methods to train and educate EMS personnel.

A sample of ambulance directors beta-tested the 23-question survey. The MDH Institutional Review Board approved the final survey tool in May 2006. The list of recipient ambulance services was obtained from the Minnesota Emergency Medical Services Regulatory Board. EMS agencies providing only air transport or facility-to-facility transport services were excluded because this survey focused on ground transport issues.

In June 2006, the survey tool, along with a cover letter written jointly by the MSP and MDH, was sent to the 240 directors overseeing all 257 ambulance services in the state. Respondents were given the option to complete the survey online. Two follow-up letters were sent to ambulance services not responding within 6 weeks. A total of 199 (77% response rate) surveys were completed. The survey closed in August 2006. A copy of the survey is available at http://www.health.state.mn.us/cvh.

### Minnesota Acute Stroke Treatment System Survey

MSP members with expertise in clinical stroke care developed the survey of hospitals. Questions were based on recommendations from the Brain Attack Coalition for stroke centers (5) and from the American Stroke Association Stroke Systems of Care (4) planning committee for Minnesota. The MDH Institutional Review Board approved the final survey of 22 questions in October 2005. The list of recipient hospitals was obtained from the MDH Facility and Provider Compliance Division Web site (www.health.state.mn.us/divs/fpc/fpc.html). The survey excluded hospitals that serve primarily children or otherwise are specialty care facilities (e.g., rehabilitation hospitals) because this assessment focused on adults receiving acute care.

Every hospital was contacted by telephone to determine the best recipient of the survey. Recipients were mostly directors of nursing or quality improvement. The survey, along with a cover letter written jointly by MSP and MDH, was sent to 133 acute care hospitals in January 2006. Respondents were given the option to complete the survey online. Two follow-up letters were sent to hospitals not responding within 6 weeks. Hospitals that did not return the second follow-up survey were contacted by telephone to conduct the survey. A total of 120 (90%) of 133 hospitals responded. Nineteen hospitals within the seven-county Minneapolis–St. Paul metropolitan area ("Twin Cities metro area") responded, compared with 101 rural (non-metro and small metro area) hospitals. Of the 13 hospitals not responding to the survey, 11 reported fewer than 100 beds, and 11 are located outside the Twin Cities metro area. The survey closed in April 2006. A copy of the survey is available at www.health.state.mn.us/cvh.

### Data analysis

Data for both surveys were analyzed by size of hospital (according to number of licensed beds) and location (Twin Cities metro and rural). Hospitals were grouped into the following categories: small (<50 beds [n = 75]), medium (50–99 beds [n = 17] and 100–249 beds [n = 9]), and large (≥250 beds [n = 19]).

## Results

### Minnesota Emergency Medical Services Stroke Care Survey

**Stroke patient transportation and advance notification**

Fewer than two-fifths (37%) of respondents reported the existence of a written transportation protocol for stroke patients (Table 1). Most (91%) would not bypass a hospital to send a patient to a destination with greater capacity for stroke care; according to written comments, ambulances take patients to a hospital chosen by the patient or patient's family. Most ambulances (78%) provide advance notice to hospitals about stroke patients needing urgent care, with notification on scene (10%), immediately en route (21%), or as soon as possible en route (54%); the questionnaire did not define the difference between "immediately" and "as soon as possible." Ten percent of ambulance services alert the destination hospital within 5 minutes before reaching it. Air transport is used infrequently; 70% never use a helicopter.

**Prehospital care and management**

Seventy-six percent of services have a written protocol for prehospital stroke management (Table 2). The Cincinnati Stroke Scale (5) (or some modification of it) is the most commonly used stroke scale assessment tool. The stroke scale score is reported to hospital staff "sometimes" in 21% of cases, "always" in 34% of cases, and "rarely" or "never" in 36% of cases; 9% of respondents did not know whether the stroke scale score is reported to hospital staff.

**Stroke training for EMS**

A majority of respondents reported their personnel are trained on stroke at least once a year (119 [60%]) (Table 3). Nearly one-third (60 [30%]) reported their personnel receive stroke training once every 2 or 3 years. Classroom training was the most commonly cited format, followed by DVDs or videos (Table 3). Other types of training reported include run reviews and Advanced Cardiac Life Support refresher courses (6). Most respondents reported offering training opportunities annually (56%) or semiannually (24%) is most effective.

Forms of training reported most often as most effective include agency-sponsored conferences and seminars, regional conferences, Internet-based training, and statewide conferences (Figure). Agency-sponsored conferences or seminars was the kind of training most commonly reported effective for Emergency Medical Technician (EMT)-Basic (79%) and, to a lesser degree (32%), for paramedics. This form of training also was reported as effective for paramedics (32% of responses). Internet-based training was the second most common method for paramedics (22%), with regional (18%) and statewide (16%) conferences cited about equally. Teleconferences, videoconferences, and national conferences were least frequently cited as effective training vehicles.

**Figure.**
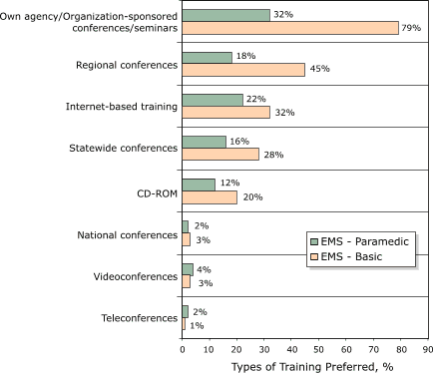
Percentage of types of training deemed most effective by emergency medical services (paramedic and basic) staff, Minnesota Emergency Medical Services Stroke Care Survey, 2006. Respondents were instructed to indicate (by checking) as many types as they wished.

**Table:** 

### Minnesota Acute Stroke Treatment System Survey

**Emergency departments**

Eighty-four percent of the largest hospitals reported having written emergency department (ED) care protocols for acute stroke diagnosis or treatment, compared with 43% of small hospitals (Table 4). Fifty-two percent of rural hospitals have ED protocols. Of the hospitals with written stroke care protocols, 10 (17%) use telemedicine.

ED staff in approximately 80% of medium and large hospitals receive training for acute stroke diagnosis and treatment, compared with 60% of small hospitals and 65% of rural hospitals. Most of the hospitals with a stroke team are based in the Twin Cities metro area. One hundred twelve (93%) hospitals reported the ability to provide around-the-clock laboratory services, with expedited results within 45 minutes.

Among the rural hospitals, 16% do not have computerized tomography (CT) scan or a CT technician available around the clock (Table 4). Of the 17 hospitals that do not have access to a neurologist for consultation on stroke cases, all but one were rural hospitals; four hospitals reported having access to a neurologist for consultation during specific hours of the day.

**Inpatient care**

Approximately 27% of small, 58% of medium-sized, and 35% of rural hospitals have written inpatient protocols for managing stroke patients (Table 5). Fewer than 20% of small hospitals have a "stroke code" (alert system for hospital staff to respond to an inpatient stroke). Most (56) of the 60 hospitals that do not provide coordinated stroke care have fewer than 100 beds. Nearly every hospital that indicated not providing such coordinated stroke care reported being prepared to transfer stroke patients to an appropriate hospital for such care. Eight percent of rural hospitals, compared with 68% of Twin Cities metro hospitals, have a designated ward for stroke patients.

**Quality improvement, education, and primary stroke center certification**

At the time of the survey (January 2006), the Joint Commission had certified six Twin Cities metro hospitals as Primary Stroke Centers.

Forty-four (37%) hospitals reported their staff had sufficient opportunity to receive at least 8 hours each year of stroke education and training (Table 6). Staff in 10 (16%) of the 63 smallest hospitals have such an opportunity. About half of hospitals with 100 or more beds provide public education; 7% of small hospitals and 31% of medium-sized hospitals provide public education on stroke at least twice a year. Twenty percent of small hospitals and 42% of medium-sized hospitals have a database for tracking quality information. One-fourth of hospitals reported having a stroke champion working within the hospital as an advocate for improving stroke care.

Hospitals were asked to name their priorities for stroke, and written responses were grouped (Table 7). Development of protocols, standing orders, or pathways (31% overall) were most commonly reported, followed by early identification or assessment of stroke patients (20%) and staff education on stroke (19%). Other responses included rapid intervention or use of tissue plasminogen activator (tPA) (14%) and rapid transfer of stroke patients (13%) (Table 7).

**Stroke hospitalizations and tPA questions**

Seventy-two (61%) of 118 hospitals reported administering intravenous tPA for acute stroke treatment. Among the 74 small hospitals, 35 (49%) administer tPA, compared with 19 (73%) of 26 medium hospitals and 17 (89%) of 19 large hospitals. Among rural hospitals, 56 (56%) of 99 administer tPA, compared with 17 (89%) of 19 Twin Cities metro hospitals.

## Discussion

The Minnesota Emergency Medical Services Stroke Treatment Survey 2006 provided valuable information about current opinions on stroke, transport and treatment practices, and training format preferences for Minnesota ambulance service personnel. For example, few services reported using a helicopter to transport stroke patients. Although helicopter transport may not be practical within the Twin Cities metro area because distance and traffic congestion are not significant barriers, services in rural areas should consider using air transport to reach a hospital within the window for using thrombolytic therapy.

A minority of EMS have a written policy for where to transport patients with acute stroke. Although most hospitals in Minnesota claim to be prepared to either treat or transfer stroke patients, the current practice of transporting to the nearest hospital highlights the need to increase the capacity of hospitals statewide to treat patients with acute stroke according to current clinical practice guidelines.

Respondents reported CD-ROM- or Internet-based training curricula and conferences or seminars as the most effective vehicles for training ambulance personnel. However, no further details were obtained about how effectiveness was assessed.

The Minnesota Acute Stroke Treatment System Survey 2006 identified many areas of strength in stroke care. These include around-the-clock laboratory services and CT scan services, plans for transferring stroke patients in hospitals without coordinated post-ED care, and ability to administer intravenous tPA for treatment of acute stroke. However, much work remains to build an effective and efficient system of care for stroke patients in Minnesota hospitals.

At the time of the survey, only six hospitals had been certified as Primary Stroke Centers; since then, three additional hospitals have been certified. The Joint Commission is an independent, nonprofit organization that accredits and certifies health care organizations and programs in the United States. The Joint Commission Primary Stroke Center certification program is recognized nationwide as a symbol of quality that reflects an organization's commitment to meeting certain performance standards.

Limitations of both surveys should be considered in interpreting results. First, accuracy of responses was limited to respondents' knowledge about stroke practices and capacity. The extent to which respondents queried more informed colleagues to answer questions for which they were unsure is unknown. In addition, self-reported information has the potential for bias toward positive answers when the opposite may be true. Moreover, none of the responses were confirmed by external review or formal inquiry. Despite their limitations, these data are valuable for planning changes and developing a coordinated system of stroke care in Minnesota.

### Implications

The MSP Steering Committee developed the following recommendations for consideration by ambulance services, hospitals, state agencies, and collaborating partner organizations in making systems-level improvements in stroke care. These suggestions and strategies were based on the results of these surveys and on discussion in quarterly meetings during 2006 after the surveys.

#### Prehospital care

Prehospital EMS personnel should continue to treat stroke as an emergent event.

Because a large proportion of respondents reported that first responders typically provide only vital sign information to ambulance staff about a patient with a suspected stroke, without indicating they suspect a stroke, MSP and its partners should consider teaching first responders to recognize the signs of stroke and to communicate such information to ambulance personnel.

MSP and its partners should encourage all ambulance service organizations to use the Cincinnati Stroke Scale as the standard for assessing patients suspected of having stroke. By having all ambulance organizations voluntarily adopt the Cincinnati Stroke Scale, a common standard can be established for training and educating ambulance personnel.

MSP and its partners should inform hospital ED staff that this stroke scale standard is being encouraged, and prehospital providers should include instructions in their standard operating procedures to always verbally provide stroke scale information to hospital ED staff. This communication will potentially aid the efficiency of care provided in the destination hospital.

MSP and its partners should inform prehospital EMS providers about the locations of primary stroke centers and educate them about recent recommendations for stroke systems of care.

MSP and its partners should provide training for prehospital EMS providers on stroke issues annually or semiannually. This training should be offered, but not mandated, for ambulance personnel.

MSP and its partners should provide classroom education or regional conferences and seminars — probably the most effective methods for education and training. Internet-based training also should be provided as an option for continuing education.

#### Emergency department

Hospitals that do not currently have standing orders for treating acute stroke in the ED should develop and share protocols. The state agency (MDH), in collaboration with partners, may be in the position to provide technical assistance to ED staff in all hospitals — but especially small and rural hospitals — to aid implementation of protocols and standing orders.

ED protocols in rural hospitals should consider including telemedicine. Relationships should be fostered between appropriate facilities (e.g., hospitals within the same health systems) to help development of telemedicine to improve acute stroke care.

#### Inpatient care

MSP and its partners should develop care protocols and pathways for inpatient care of stroke patients and share these tools among hospitals, particularly rural hospitals.

Staff at hospitals that do not have a stroke code for inpatient stroke events should be trained on developing and implementing protocols.

#### Quality improvement and education

MSP and its partners should provide technical assistance and lessons learned to all hospitals that plan to become certified as Primary Stroke Centers by the Joint Commission.

MSP and its partners should provide ongoing stroke training and continuing education opportunities for hospital staff on the following topics:

- Protocol development and implementation
- Evidence-based use of tPA
- Early and rapid assessment of stroke (National Institutes of Health Stroke Scale training)
- Updates and current practices for stroke care

Partner organizations may collaborate with hospital education and outreach departments to develop community-based stroke education efforts, particularly during May (Stroke Awareness Month).

Partners should collaborate on increasing the number of hospital EDs that administer intravenous tPA in appropriate and eligible stroke patients.

MSP and its partners should develop a voluntary hospital network, where purpose, infrastructure, and initiatives are defined and implemented by multiple partners, including the state quality improvement organization, state hospital association, state office of rural health, and the statewide coalition or task force for stroke.

### Conclusions

The results of these surveys will guide MDH and MSP in planning for systems-level intervention and programs to improve acute stroke care in Minnesota. In addition, ambulance services and hospitals can use these results to change their health systems to improve the quality of care for their stroke patients. Finally, these surveys highlight the need to develop a statewide or regional system for transportation and treatment of stroke patients to better facilitate coordination between EMS and hospital care. This system may involve development of formal or informal agreements to divert patients to appropriate facilities, establishment of a structure for providing continuing education to health care providers, and opportunities for health care organizations to improve their overall stroke care capacities.

## Figures and Tables

**Table 1:** Responses to Questions About Transportation of and Advance Notification Practices for Stroke Patients, Minnesota Emergency Medical Services Stroke Care Survey (N = 199), 2006

**Topic**	No. (%)
Strokes typically are treated as emergent (urgent)^a^ by dispatch	184 (92)
Comprehensive information from first responders^b^ typically is provided to ambulance staff upon arrival at a suspected stroke patient	7 (4)
Written policy exists to determine where patients with acute stroke are transported	73 (37)
Hospitals are bypassed to transport suspected stroke patients to hospital with more stroke care capabilities	17 (9)
Distance to destination hospital, if bypassed a closer hospital, is greater than 25 miles	99 (50)
Suspected stroke patients are received by a specialized stroke team at the hospital (based on self-report)	14 (7)
Suspected stroke patients receive immediate attention at the hospital	168 (84)
Policy exists to give hospitals advance notification of stroke patients en route	155 (78)
Emergency medical services personnel activate alert system^c^ on scene or immediately en route	62 (31)
Ambulance crew treats suspected stroke patients as urgent	175 (88)
Helicopter is used very often or often for transport	17 (9)

a Emergent (urgent), versus not emergent, is a term used somewhat congruently for whether lights and sirens are used en route to the hospital; however, use of lights and sirens is not necessary for an incident to be considered emergent.

b Defined as the first medically trained responder to arrive on scene (e.g., police, fire, emergency medical services, lay rescuer).

c The survey did not define "alert" system, but it typically is considered any system that announces that a rapid-response team at a destination hospital needs to be assembled for an incoming emergency.

**Table 2:** Responses to Questions About Prehospital Care and Management of Stroke Patients, Minnesota Emergency Medical Services Stroke Care Survey (N = 199), 2006

**Topic**	No. (%)
Written protocol exists for management of suspected stroke patient	152 (76)
Cincinnati Stroke Scale (or modified version) used by personnel	93 (47)
Personnel always verbally report stroke scale findings to destination hospital staff	68 (34)
Stroke scale data are recorded	102 (51)

**Table 3:** Responses to Questions About Emergency Management Services Stroke Training Frequency and Formats Currently Used, Minnesota Emergency Medical Services Stroke Care Survey (N = 199), 2006

**Topic**	No. (%)
Personnel are trained at least once a year on stroke	119 (60)
Classroom training on stroke offered by organization	139 (70)
DVD or videos used for training	19 (10)
Internet-based resources used for training	6 (3)
Annual or semiannual stroke education opportunities most effective	159 (80)

**Table 4:** Responses of Hospitals to Questions About Emergency Department Stroke Care Capacity, Minnesota Acute Stroke Treatment System Survey, 2006

Topic	Overall N = 120	Location		Hospital Size^a^		

		Rural n = 101	Metro n = 19	Small n = 75	Medium n = 26	Large n = 19

	No. (%)	No. (%)	No. (%)	No. (%)	No. (%)	No. (%)
Written ED care protocols (standing orders) exist for acute stroke diagnosis/treatment	68 (57)	53 (52)	15 (79)	32 (43)	20 (77)	16 (84)
ED staff receive training in care protocols for acute stroke diagnosis and treatment	81 (68)	66 (65)	15 (79)	45 (60)	21 (81)	15 (79)
Designated stroke team is available 24/7	16 (13)	4 (4)	12 (63)	2 (3)	4 (15)	10 (53)
Lab services are available 24/7 with expedited results in 45 minutes	112 (93)	95 (95)	17 (89)	70 (93)	25 (96)	17 (89)
CT scan and CT technician available 24/7	101 (84)	84 (84)	17 (89)	61 (81)	24 (92)	16 (84)
Access to neurologist is available for consultation on stroke cases	103 (86)	85 (85)	18 (95)	65 (87)	20 (77)	18 (95)
Preference exists for the method of transport of stroke patients^b^	68 (57)	62 (62)	5 (26)	47 (63)	18 (69)	3 (16)

ED indicates emergency department; CT, computerized tomography.

a Small indicates <50 beds; medium, 50–99 beds (n = 17) and 100–249 beds (n = 9); and large, ≥250 beds.

b This question refers to the preference by the ED to transport patients to another facility, whether by helicopter, ambulance, or other form of transportation.

**Table 5:** Responses of Hospitals to Questions About Inpatient Stroke Care Capacity, Minnesota Acute Stroke Treatment System Survey, 2006

Topic	Overall N = 120	Location		Hospital Size^a^		

		Rural n = 101	Metro n = 19	Small n = 75	Medium n = 26	Large n = 19

	No. (%)	No. (%)	No. (%)	No. (%)	No. (%)	No. (%)
Written inpatient protocol for management/care ("critical pathways" or "standing orders") exist for stroke patients	50 (42)	35 (35)	15 (79)	20 (27)	15 (58)	15 (79)
"Stroke code"^b^ or a process exists for rapid treatment of inpatient strokes	33 (28)	21 (21)	12 (63)	14 (19)	6 (23)	13 (68)
Hospital provides coordinated stroke care beyond emergency department physician's evaluation^c^	55 (46)	39 (39)	16 (84)	23 (31)	16 (62)	16 (84)
Designated ward exists for stroke patients	21 (18)	8 (8)	13 (68)	1 (1)	6 (23)	14 (74)

a Small indicates <50 beds; medium, 50–99 beds (n = 17) and 100–249 beds (n = 9); and large, ≥250 beds.

b A "stroke code" is an alert system for hospital staff to respond to an inpatient stroke.

c Coordinated stroke care is comprehensive care for an admitted stroke patient that is coordinated between the ward, rehabilitation services, and other health care professionals.

**Table 6:** Responses of Hospitals to Questions About Quality Improvement and Education Issues, Minnesota Acute Stroke Treatment System Survey, 2006

Topic	Overall N = 120	Location		Hospital Size^a^		

		Rural n = 101	Metro n = 19	Small n = 75	Medium n = 26	Large n = 19

	No. (%)	No. (%)	No. (%)	No. (%)	No. (%)	No. (%)
Hospital certified by Joint Commission as a Primary Stroke Center	6 (5)	0 (0)	6 (32)	0 (0)	2 (8)	4 (21)
Hospital meets Brain Attack Coalition recommendations for a primary stroke center	5 (4)	4 (4)	1 (5)	4 (5)	0 (0)	1 (5)
Staff have sufficient opportunity to receive at least 8 hours of continuing stroke education annually	44 (37)	28 (28)	16 (84)	10 (13)	8 (31)	16 (84)
Hospital presents a minimum of two programs per year educating the public on stroke risk factor reduction and signs and symptoms of acute stroke	24 (20)	12 (12)	12 (63)	5 (7)	8 (31)	11 (58)
Database or system exists to collect data and to track quality improvement activity related to stroke patients	40 (33)	26 (26)	14 (74)	15 (20)	11 (42)	14 (74)
Hospital has a stroke champion	31 (26)	15 (15)	16 (84)	7 (9)	8 (31)	16 (84)

a Small indicates <50 beds; medium, 50–99 beds (n = 17) and 100–249 beds (n = 9); and large, ≥250 beds.

**Table 7:** Summary of Key Priorities of Surveyed Hospitals , Minnesota Acute Stroke Treatment System Survey, 2006

Topic	Overall N = 120	Location		Hospital Size^a^		

		Rural n = 101	Metro n = 19	Small n = 75	Medium n = 26	Large n = 19

	No. (%)	No. (%)	No. (%)	No. (%)	No. (%)	No. (%)
Development of protocols, standing orders, or pathways	37 (31)	30 (30)	7 (37)	24 (32)	8 (31)	5 (26)
Early identification or assessment of stroke patients	24 (20)	20 (20)	4 (21)	17 (23)	4 (15)	3 (16)
Staff education on stroke	23 (19)	20 (20)	3 (16)	14 (19)	6 (23)	3 (16)
Rapid intervention or tPA use	17 (14)	13 (13)	4 (21)	8 (11)	5 (19)	4 (21)
Rapid transfer of stroke patients	15 (13)	15 (15)	0 (0)	13 (17)	2 (8)	0 (0)
Community education on stroke	11 (9)	11 (11)	0 (0)	8 (11)	2 (8)	1 (5)
Improvement of CT or MRI capabilities	10 (8)	10 (10)	0 (0)	10 (13)	0 (0)	0 (0)
Joint Commission certification	9 (8)	2 (2)	7 (37)	0 (0)	1 (4)	8 (42)
Coordination of care	8 (7)	5 (5)	3 (16)	5 (7)	2 (8)	1 (5)
Quality improvement (general)	7 (6)	6 (6)	1 (5)	0 (0)	2 (8)	5 (26)
Patient education on stroke	6 (5)	4 (4)	2 (11)	3 (4)	1 (4)	2 (11)
Quality of care through the continuum, including rehabilitation	6 (5)	5 (5)	1 (5)	3 (4)	1 (4)	2 (11)
Development and/or marketing of the stroke team	4 (3)	4 (4)	0 (0)	1 (1)	3 (12)	0 (0)
Other	5 (4)	3 (3)	2 (11)	2 (3)	0 (0)	3 (16)

tPA indicates tissue plasminogen activator; CT, computerized tomography; MRI, magnetic resonance imaging.

a Small indicates <50 beds; medium, 50–99 beds (n = 17) and 100–249 beds (n = 9); and large, ≥250 beds.
